# Cushing’s disease and bipolar disorder: study of medicopsychiatric intricacies from a clinical vignette

**DOI:** 10.1192/j.eurpsy.2023.1460

**Published:** 2023-07-19

**Authors:** F. Z. Chamsi, H. Abrebak, T. amine, A. El ammouri

**Affiliations:** psychiatry department, university hospital tangier morocco, tangier, Morocco

## Abstract

**Introduction:**

Cushing’s syndrome is a relatively rare condition that results from chronic hypercortisolism. This syndrome is characterized by the presence of various psychiatric manifestations that can accompany it at all its stages of evolution. They can either inaugurate the clinical picture, or appear during the course of the disease, as they can persist even after the resolution of the syndrome. of Cushing. Through a clinical vignette, we report a case of Cushing’s syndrome in a 52-year-old patient who presented with a picture of melancholic depression as part of a bipolar disorder and who had preceded the discovery of Cushing’s disease.

**Objectives:**

establish the relationship between Cushing’s syndrome and bipolar disorder and identify the main pillars of care.

**Methods:**

It was proposed to present the clinical case of a 52-year-old patient in whom psychiatric manifestations of the psychotic and thymic types preceded the discovery of Cushing’s disease, to recall the main psychiatric symptoms that can be encountered during such endocrine disorders and their possible entanglement with psychiatric pathologies.

**Results:**

Mrs. F. is 52 years old. married and mother of 3 children, without social security coverage for medullary carcinoma of the thyroid, having benefited from thyroidectomy with radiotherapy, admitted for treatment of paraneoplastic Cushing’s syndrome. The beginning of his troubles goes back to 6 months before his consultation with psychiatry. The initial symptoms were typical of a progressive weight gain noticed by the entourage. There was the installation of thymic symptoms such as an elation of mood, self-esteem, multiple projects. One month before admission to the internal medicine department, the patient presented with a depressed mood, anhedonia , a disgust for life, and dark thoughts. She had stopped all drug treatment.

Cushing’s disease is the most common cause of endogenous hypercortisolism (>85%). Corticotropic micro-adenomas , are the most frequently observed, i.e., in 90% of cases, and are sometimes not visible on magnetic resonance imaging . Its first-line treatment is neurosurgical with trans-sphenoidal excision. In its second intention, it uses drugs whose targets are pituitary or adrenal, as well as radiotherapy.

**Conclusions:**

we present the case of a patient with a picture of bipolar mood disorder and Cushing’s disease. Thymic and psychotic psychiatric symptoms preceded the discovery of endocrine disease whose symptoms were not apparent at first. The hypotheses of comorbidity or rather of the inauguration of the endocrine disease by a psychiatric picture, in particular bipolar mood disorder, remain advanced and the limits between

**Disclosure of Interest:**

None Declared

